# The Trend of Research in Mental Hospitals

**Published:** 1914-01-24

**Authors:** 


### MEDICO-PSYCHOLOGY.

### The Trend of Research in Mental Hospitals.

In regard to investigations into the causation of insanity and the psycho-neuroses, it seems as if we have come to a parting of the ways. Is the medico-psychological research of the future to rely chiefly on ordinary pathological technique and its findings to be expressed in terms of morbid anatomy? Or is it to follow the new paths opened up by the development of psychology into a recognised science and to express its results in terms of mental processes? There are some alienists who think that enough money has been spent on asylum pathological departments, and that, considering the paucity of the results derived from the *post-mortem* examination of thousands of brains, it is not to be anticipated that much progress will result from further work of this kind. Such a pessimistic attitude towards the future of asylum pathology is, however, by no means justified. Although, as compared with the advances made in general systemic disease, pathology has but slightly increased our understanding of mental disorder; and, indeed, the splendid developments of material science during the past fifty years have helped us very little, if at all, towards the elucidation of the problems as to why men should be seized by delusions, hallucinations, morbid excitement, and vain imaginings. Nevertheless, pathological methods have enormously advanced our knowledge of insanity in one particular direction at least.

For researches such as have been carried out under the direction of Dr. F. W. Mott, in the laboratories of the London County Council at Claybury Mental Hospital, and by Dr. Shaw Bolton, of the West Riding, whose work in the histo-pathology of amentia and dementia is classical, have clearly demonstrated that very often mental disease is really dependent on morbid brain changes. This has, of course, in some sense been understood for many years from ordinary *post-mortem* work, but only comparatively lately has any scientific analysis of observed phenomena been attempted. This, during the last twenty years' work of this kind, has resulted in the accumulation of a vast amount of data showing the effect of organic intracranial disease on the workings of the mind. Particularly has it been shown to what an appalling extent alcoholism and venereal disease are responsible for premature breakdown of the organ of mind, and consequent mental derangement. Year by year the pathological laboratories of the institutions are adding to our knowledge of the ravages in the brain caused by tuberculosis and infective conditions of its covering membranes, as well as by new growths; making it more and more easy for the general practitioner to understand how far disturbance of mind may be due to physical processes of this kind. And, although these investigations have not made clear the cause of many forms of insanity, they have led to the very satisfactory result that whilst comparatively few years ago the physician had to confront a seemingly hopeless puzzle whenever he was called in to a case of insanity, the medical man of to-day is able to proceed on definite lines, and, as a first step, ascertain by means of various physical signs whether organic brain disease is present in any particular case. In many instances of mental trouble systematic examination of this kind reveals the presence of meningitis, glioma, tuberculides, syphilides, or degenerations; all conditions serious enough, but for which, nevertheless, modern methods of treatment offer hope of relief in a certain percentage of cases. Such systematic reports as the "Archives of Neurology," issued from the Claybury laboratories, are veritable mines of information respecting the effects of organic brain lesions in the production of mental symptoms. The results obtained by microscopical examination of the cerebral blood vessels alone justify the further developments of facilities for asylum research. Particularly instructive have been those investigations which have demonstrated the disastrous consequences of various infections—notably syphilis and tuberculosis—as well as of degenerative processes on the arteries of the brain. Then, again, in one or two well-defined diseases—for example general paralysis of the insane—histo-pathological research has most clearly revealed the disastrous physical consequences in the brain, although there may still be disagreements in regard to the causative organisms.

Evidently, then, the results of organised research on the lines of morbid anatomy followed in the *post-mortem* departments of the general hospitals have been productive of excellent consequences; such excellent results, indeed, that still more important advances may be confidently awaited from further research in this direction. On the other hand, there remain many types of insanity and forms of psycho-neurosis concerning which pathologists have so far taught us nothing at all; and there is a growing tendency to attack the problems thus presented as problems of mental processes and not of organic conditions. Of the nature of mind we know nothing in the scientific sense, but we do know that mental processes are amenable to classification and analysis; whilst, furthermore, they are open to influences of will, not only the self-command of the individual over his own thoughts, but to outside mind influence. The development of medico-psychological research on these lines represents one of the most interesting departures of late years, and the study of the processes of what may be termed "morbid thought" in their relation to suggestion and their amenability to accurate analysis is to-day occupying the attention of an ever-increasing body of responsible workers. Practically, then, it seems clear that every institution should provide its staff with adequate facilities for research according to the principles of each school of research.

